# Insomnia, excessive daytime sleepiness, anxiety, depression and socioeconomic status among customer service employees in Canada

**DOI:** 10.5935/1984-0063.20190133

**Published:** 2020

**Authors:** Faustin Armel Etindele-Sosso

**Affiliations:** 1 Hôpital du Sacré-Coeur de Montréal, Center for Advanced Research in Sleep Medicine (CEAMS) - Montréal - Qc - Canada; 2 Université du Québec à Montréal, Institut Santé et Société - Montréal - Qc - Canada; 3 Douglas Institute of Mental Health, Quebec Network on Suicide, Mood Disorders and Related Disorders (RQSHA) - Montréal - Qc - Canada

**Keywords:** Insomnia, Sleepiness, Anxiety, Depression, Socioeconomic Status, Customer Service

## Abstract

**Objective:**

It is the first study investigating deeply symptoms of neuropsychiatric diseases among a large population of customer service employees (n=1238, 640 females and 598 males). The study’s goals were document presence of sleep disorders, anxiety and depression among customer service advisors and determine the influence of the socioeconomic status (pSES), duration in position and full-time or part-time shift on the diseases above.

**Methods:**

Linear regressions and ANOVA with a Tukey multiple comparisons of means was performed to analyze correlation and differences between citizens, international students and immigrants in their pSES and neuropsychiatric diseases.

**Results:**

Customer service employees (578 Canadians, 264 immigrants and 358 international students) are in majority undergraduate students (286 men and 289 females) with a high school degree (280 men and 308 women). They work full-time (560 men and 548 women) and are single (420 men and 560 women). Among customer service advisors, the time spent as an advisor was an excellent predictor of insomnia, sleepiness and anxiety (respectively with R^2^=91,83%, R^2^=81,23% and R^2^=87,46%) but a moderate predictor of depression (R^2^=69,14%). The pSES was a moderate predictor of sleep disorders (respectively R^2^=62,04% for insomnia and R^2^=53,62% for sleepiness) and strongly associated with anxiety and depression (R^2^=82,95% for anxiety and R^2^=89,77% for depression).

**Discussion:**

Insomnia, sleepiness and anxiety are more prevalent for full-time employees (higher for immigrants and international students compared to Canadians) compared with part-time employees, while depression was similarly higher for Canadian and immigrants compared to international students. Regarding full-time employees, symptoms of insomnia, anxiety and depression were higher for men compared to women. Regarding part-time employees, symptoms of insomnia and sleepiness were higher for women compared to men. Employees working full-time with rotating shifts are more exposed to insomnia, sleepiness and anxiety than employees working part-time. More research is needed to understand mental health of customer service employees regardless of their area and it is worthy of interest to study the link between sleep disorders and mood disorders with work conditions. Here some practical suggestions are made to reduce neuropsychiatric disorders for customer service employees or to at least mitigate the work burden on their brains.

## INTRODUCTION

Nowadays, accumulating literature suggests that our modern lifestyle is accompanied by many stressors such as night work, continued flow of news via social network, a low socioeconomic status complicating healthcare or, more often, successive bad experiences leading to anxiety or depression^1^^,^^2^. An important part of this modern age is the possibility to complete almost everything online or by phone. Even if this evolution makes our lives easier, evidence suggests that cell phones have a negative impact on our physical health, socialization and brain^3^^-^7. Many studies reported associations between lack of physical activity and obesity^3^^,^^8^. It has also been shown that there is a link between poor and moderate physical activity and cardiovascular diseases^6^^,^^9^, as well a connection between development of cognitive impairment and an excessive use of technology^10^.

The impact of this lazy lifestyle on the consumer’s body has now been well documented, but there is little to no data available regarding the health of a few main actors of this new age: the advisors. Whichever field one might work in, be it shopping, technical support, shopping, need technical support, ordering, or just seeking some help or information; there are human beings behind the screen or the phone. Recent research in chronobiology and sleep medicine reported incidence of the effect of some type of work on mental health^11^^,^^12^. For example, night shift workers and pilots are exposed to shift work disorders after transitioning to rotating shifts^13^^,^^14^. Nurses and rotating shift workers who have constant flux in their shifts face a permanent disturbance of their circadian system, experience premorbid psychobiological processes as well as work-related depression and anxiety^14^. 

Owing to the permanent flux of rotating shifts in these types of jobs, work-related sleep-wake schedules often conflict with internal circadian rhythms^14^. Sleep-wake impairments in response to a circadian challenge are highly variable from an individual to another, but an important proportion of shift workers cannot synchronize their circadian clocks to their work-related sleep-wake schedules. Indeed, up to 26% of rotating shift workers develop shift work disorder, which is characterized by persistent and severe sleep disturbance during the sleep period and/or excessive sleepiness during the wake period^13^^,^^14^.

Among professions with shift work, there is customer service in call centers. The 2018 Call Center Industry Report, which is an annual report data from 750 customer service professionals from 89 countries, revealed that customer service can impact up to 30% of the local economy of few countries like India and Tunisia, and was associated with several sick leaves (2018 Call Center Industry Report). Even if the automatization of communication may decrease the call center staff in the future, human advisors remain the heart of this industry. The area of customer service is remarkably similar to those of nursing and night shift workers in terms of rotating shifts, night shift work and volume of services the advisors have to provide. These employments share characteristics like constant pressure to reach a certain objective and long working hours. This leads to several mental health issues and a general decrease of occupational health in workers from these areas^15^^,^^16^.

A recent meta-analysis by Wong et al.^17^ revealed that long working hours negatively affects the occupational health of workers, particularly in terms of sleep, mental health and physical health. Seeing that there is a link between long working hours and a decrease in physical and psychological health, it is probable that people working in customer service may suffer from neuropsychiatric diseases. The hypothesis of this research is further supported by the fact that people working jobs involving rotating shifts, such as nurses and pilots, suffer from sleep-work disorders and mood disorders.

The present paper is original in its conceptualization. The problem is the fact that this topic was not deeply investigated for customer service advisors, perhaps not explored at all. This was confirmed by the difficulty to find literature on this subject in the most popular databases such as PubMed, Scopus and Web of Science. Currently, there is no data on the prevalence of sleep disorders or what kind of sleep disorders customer service advisors experience during their shifts work or because of this job. Similarly, there is no literature on mood disorders related to working in customer service. The present paper is an exploratory study which has as objectives to: (1) document the presence of sleep disorders among customer service advisors; (2) document the presence of anxiety and depression in this population; and (3) determine the influence of socioeconomic status, duration in position and the effect of full-time versus part-time shifts on the diseases listed above.

## METHODS

### Ethical Statement

This project was approved by the Faculty of Arts and Science of the Université de Montreal (CERAS-2015-16-194-D), and secondly by the team leaders or human resources representatives of all the call centers and customer services which provide advisors. Data were collected and computed in an external database. This database was secured outside the public network and was shared only with the investigators involved in the project.

### Participants and procedure

The customer service call centers employing shift workers were contacted during the national career event and the national job fair, which both took place in three Canadians cities (Montreal, Laval and Longueil) in 2016 and 2017. These events are organized every year to allow people to find new jobs and to meet with employers who have available positions. Study objectives and protocol were explained to the representatives on-site and an electronic link to complete the study online was provided during the event. Two reminders to participate in the study were sent by email (two days and one week after the event) to the representatives we met. This allowed us to verify that their companies’ managers approved the study and shared our electronic link with their customer service advisors. A total of 52 customer services were contacted during the events above, but only 11 customer services confirmed their participation in the study (response rate of 21.15%). All participants (n=1238, 640 females and 598 males) received the study link from their administration (from their team leaders or supervisors at work). Two contact numbers and email addresses were provided to reach our research team in the first page of the online study. The contact to our ombudsman was also provided in the first page, following a description of the study in French and English. 

The online questionnaire included a consent statement related to data protection and the understanding of the study objectives, which required an acceptance and willingness to continue with the anonymous online survey by clicking “I agree”; if the participants wished to move forward with the study. The average time to answer all questions was estimated during our final testing to be thirty minutes. The questionnaire was configured in such way that it could only be completed once by participants with the same email address. Responses were collected between November 2016 and October 2017. Duplicates and incomplete forms were removed (n=38). The final sample analyzed was n=1200 participants/advisors (610 females and 590 males).

### Measures

***Insomnia Severity Index (ISI):*** This questionnaire is widely used to assess insomnia and associated parameters, including lack of sleep and sleep quality. It was developed by Bastien et al.^18^, in 2001, and widely validated and used for different populations and contexts^19^. The internal consistency of the ISI was excellent (Cronbach’s α=0.92), and each individual item showed adequate discriminative capacity (r=0.65-0.84). The area under the receiver operator characteristic curve was 0.87 and suggested that a cut-off score of 14 was optimal (82.4% sensitivity, 82.1% specificity and 82.2% agreement) for detecting clinical insomnia. An ISI score between 0 and 7 is an insomnia considered clinically non-significant, between 8 and 14 as a subthreshold insomnia, between 15 and 21 as a moderate clinical insomnia, and between 22 and 28 like as a severe clinical insomnia^18^^,^^19^.

***Epworth Sleepiness Scale (ESS):*** The Epworth Sleepiness Scale is a simple and reliable instrument that has been used worldwide since 1991. This test was developed by Dr. Murray Johns of Epworth Hospital in Melbourne, Australia^20^. This test assesses drowsiness levels throughout the day, also known as excessive daytime sleepiness. This test also indicates if a certain degree of drowsiness may warrant a visit to the doctor and a more thorough assessment to diagnose a sleep respiratory disorder. The test consists of eight questions (0 to 3 points) and is completed in less than five minutes. If the participant scores 9 points or fewer, it is considered normal. If the participant scores more than 10 points, an appointment should be made with a sleep specialist. The Epworth scale only quantifies subjective drowsiness (interpreted by the participant) regardless of its association with a sleep disorder.

***The Hospital Anxiety and Depression Scale (HADS):*** This is a self-administered test that involves a scale with 14 items, divided into two subscales of seven items (Anxiety or HADS-A, Depression or HADS-D). It contains no somatic items that can be confused with the symptomatic manifestations of a disease. Each item is scored on a scale of 0 to 3. A score is generated for each of the two sub-scales and for the entire HADS (HADS-T). The scoring is similar for anxiety and depression and following scores, there are three categories of symptomatic levels: non-cases or asymptomatic ones (scores ≤ 7); probable or borderline cases (scores 8-10); clearly or clinically symptomatic cases (scores ≥ 11). The duration of administration is approximately five minutes and psychometric properties are detailed in previous studies^21^^,^^22^.

***The MacArthur Scale of Subjective Social Status:*** This scale measured in this study the perceived Socioeconomic Status (pSES) with *the MacArthur Scale of Subjective Social Status.* This scale allows a self-report of pSES using the general socioeconomic markers such as income, occupation and education^23^^,^^24^. The online form showed participants a ladder with a number on each step, ranging from 1 to 10 (lowest to highest). Each participant was asked to choose a step of the ladder corresponding to their current feeling about their social status at this level in their lives. Participants in the present study endorsed values ranging from 2 to 7. Previous studies demonstrated that pSES is an excellent predictor of health outcomes even after adjustment for objective SES measures^23^^,^^24^.

In addition to sociodemographic data like age, marital status, immigration status (citizen, international student or immigrants), education, duration in position (ranging from 1 month to 24 months in the sample) and sex, participants were classified in full-time workers (from 30h/week to 40h/week) and part-time workers (from 20h/week to 29h/week); according to Canadian laws. The choice to dichotomize population in two groups (full-time workers and part-time workers) was made in order to precise profile of mental health trends for these two types of workers, because whatever shift work an employee has (for example student schedule, single mother or father with children; medical exemption); he/she will fall into one of these two categories.

### Data analysis

The normal distribution of the data was analyzed using a Shapiro Wilk test. Unpaired sample t-tests were used to compare difference in insomnia, sleepiness, anxiety and depression for male vs. female; and for part-time workers vs. full-time workers. First, linear regression analyses were used to investigate whether there was association between *insomnia, sleepiness, anxiety, depression* as dependent variables; and *duration in position* as an independent variable. Next, linear regression analyses were used to assess if *insomnia, sleepiness, anxiety and depression* could be predicted with *perceived socioeconomic status* as an independent variable. Finally, a one-way ANOVA with a Tukey multiple comparisons of means was performed to analyze differences between citizens, international students and immigrants in their level of anxiety, depression, insomnia and excessive daytime sleepiness. For the exploratory aims of this cross-sectional study, the immigration status was considered more sensitive for an accurate description of the population working in customer service using the ANOVA. Even if education and income may be interesting to assess the socioeconomic status of workers, they were not appropriated for the ANOVA because immigrants and international students have educational degrees which are often considered lower or higher than degrees with same names in Canada; and income is not a good indicator because some people have more than one job and are thus difficult to rank in a category (low income, middle income, etc.). All analysis was controlled for job/occupation in the company (manager, advisor, human resources agent, etc.), for age, for income, postal code of home and sex. All non-significant analysis was not reported. All the statistical tests used an alpha of 0.05 as level of significance. Data analysis was performed using PRISM (Graph Pad Prism, version 7.0.0.159, Graph pad software) and SPSS version 22 (IBM Corp, Armonk, NY, USA)

## RESULTS

### Profile of the population working in customer service

Table 1 presents the descriptive data and the sociodemographic characteristics of the sample. The sample is almost identical in proportion of men and women (respectively, 49.17% and 50.83%), with most of them having a secondary/high school degree (47.45% for men, 50.5% for women) and an undergraduate diploma (48.47% for men, 47.37% for women). The Majority of customer service employees in this population were full-time workers (94.82% for men and 89.84% for women). The majority of customer service employees reported a marital status “single” (71.18% for men and 91.8% for women), while 22.71% of men were married compared to only 6.23% of women. In the sample, almost half were Canadian-born (44.07% of men and 52.13% of women) while the other half of workers were immigrants and international students. It was found that in men, 30.51% were international students (both with permits of study or permits of work) and 25.42% were immigrants (those with the status of permanent residents or refugees). Similar trends were found for women with 13.77% of international students and 34.1% of immigrants.

**Table 1:** Sociodemographic characteristics of the population working in customer service

	Men	Women	*p*-Value
Number (n, %)	590 (49,17%)	610 (50,83%)	0.001
Mean age ± SD	26 ± 4	21 ± 2	0.001
Number of part-time workers -20h/week to 29h/week- (n,%)	30 (5,08%)	62 (10,16%)	0.001
Number of full time workers -30h/week to 40h/week- (n, %)	560 (94,82%)	548 (89,84%)	0.001
Average income for part-time workers (CAD$)	18360	18430	0.001
Average income for full-time workers (CAD$)	31500	30600	0.001
**Education/degree (%)**			
*Secondary/high school	280 (47,45%)	308 (50,5%)	0.001
*Undergraduate studies (Bachelor's, Cegep, certificate, etc…)	286 (48,47%)	289 (47,37%)	0.01
*Graduated studies (Master's)	24 (4,07%)	13 (2,13%)	0.001
*Graduated studies (PhD, Postdoctorate, MBA, etc…)	0	0	
**Marital Status (n, %)**			
Single	420 (71,18%)	560 (91,8%)	0.001
Divorced	36 (6,1%)	12 (1,97%)	0.01
Married	134 (22,71%)	38 (6,23%)	0.001
**Citizenship**			
Canadian-born	260 (44,07%)	318 (52,13%)	0.001
International students (with permit of study and permit of work)	180 (30,51%)	84 (13,77%)	0.001
Immigrants (permanent resident, refugees)	150 (25,42%)	208 (34,1%)	0.001

Distribution of insomnia, excessive daytime sleepiness, anxiety, depression and pSES between full-time workers and part-time workers male and female

**Figure 1:**
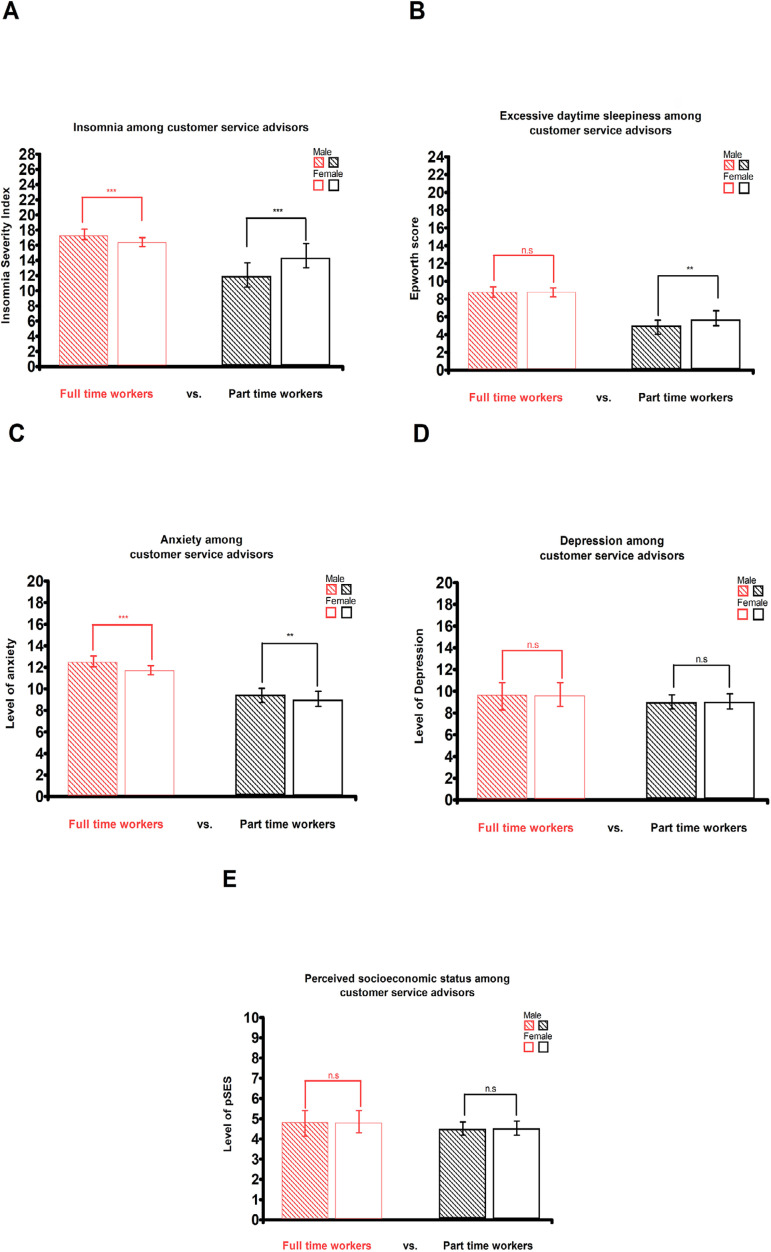
**Distribution of neuropsychiatric disorders in the sample. A: Differences in insomnia’s level among customer service advisors**. Insomnia’s level is the mean of individuals score on the Insomnia Severity Index (ISI) for both men vs. woman and full time workers vs. part time workers. **B: Differences in Excessive daytime sleepiness among customer service advisors**. Drowsiness’s level is the mean of individuals score on the Epworth Sleepiness Scale (ESS) for both men vs. woman and full time workers vs. part time workers. **C: Differences in anxiety among customer service advisors**. Anxiety’s level is the mean of individuals score on the Hamilton anxiety scale (HADS-A) for both men vs. woman and full time workers vs. part time workers. **D: Differences in Depression among customer service advisors**. Depression’s level is the mean of individuals score on the Hamilton depression scale (HADS-B) for both men vs. woman and full time workers vs. part time workers. **E: Differences in socioeconomic status among customer service advisors**. The pSES (perceived socioeconomic status) is based on the mean of individuals score on the MacArthur Scale of Subjective Social Status, for both men vs. woman and full time workers vs. part time workers. Differences were tested with paired t-tests. **Legends.** *p* < 0.0001 (***), *p* < 0.001 (**), *p* < 0.01 (*), *p* ^3^ 0.05 (n.s)

Unpaired t-tests were used to assess differences in the level of insomnia, excessive daytime sleepiness, anxiety, depression and pSES between full-time workers and part-time workers; and between male and female. The Figure 1 reports these results. It was found for full-time workers an insomnia’s mean score of 17/28 for men and 16/28 for women (*p*<0.001); and for part-time workers an insomnia’s mean score of 12/28 for men and 15/28 for women (*p*<0.001). Excessive daytime sleepiness score was an average of 9/24 for both men and women working full-time (*p*>0.05), and for part-time workers it was found 6/24 for women and 5/24 for men (*p*<0.001). The Figure 1C shows an anxiety’s mean score of 12.5/20 for men and 11.8/20 for women working full-time (*p*<0.001); and 9.8/20 for men and 9/20 for women working part-time (*p*<0.001). Figure 1D shows similar mean score of 9.8/20 for both men and women working full-time (*p*>0.05), and a 9/20 for both men and women working part-time (*p*>0.05). The Figure 1E shows an average pSES of 4.9/10 for both men and women working full-time (*p*>0.05), and 4.5/10 for men and women working part-time (*p*>0.05).

**Figure 2:**
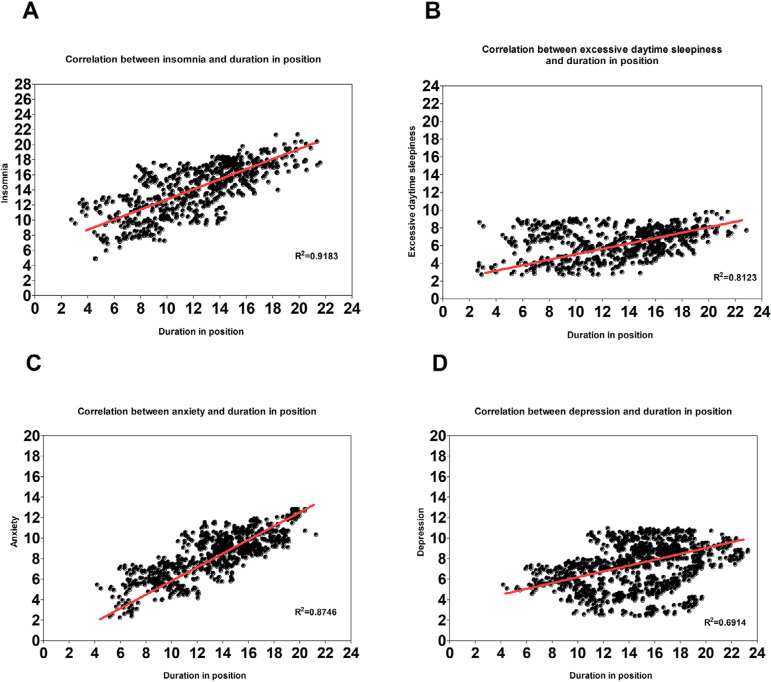
**Prediction of neuropsychiatric disorders by the duration in position**. Linear regressions were used to assess the correlation between *insomnia, sleepiness, anxiety, depression* as dependent variables; and as an independent variable. All analysis were controlled for age and sex. All the statistical tests used an alpha of 0.05 as level of significance. Confidence interval is 95%.

**Figure 3:**
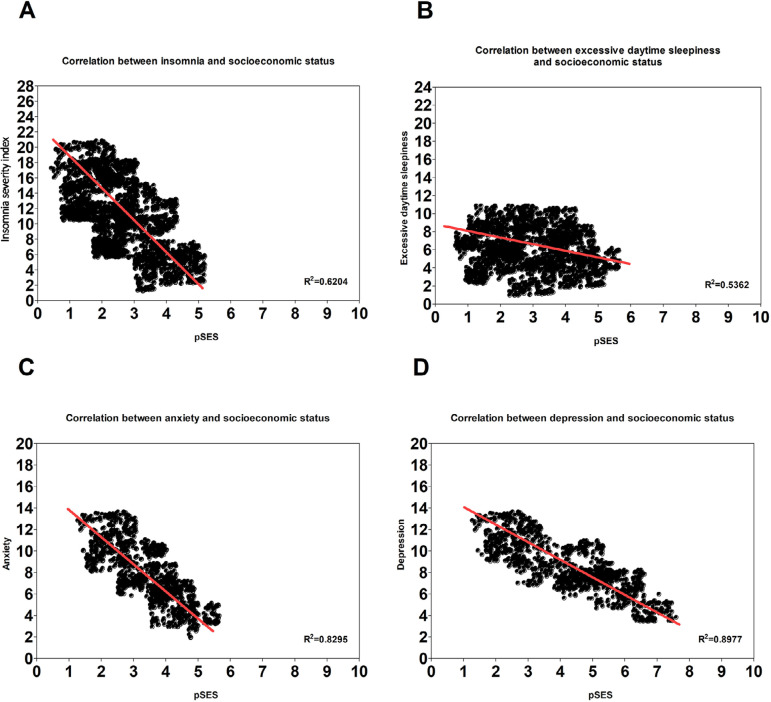
**Prediction of neuropsychiatric disorders by the socioeconomic status.** Linear regressions were used to assess the correlation between *insomnia, sleepiness, anxiety, depression* as dependent variables; and perceived socioeconomic status as independent variable. All analysis were controlled for age and sex. All statistical tests used an alpha of 0.05 as level of significance. Confidence interval is 95%.

The Figure 2 depicts the association between each neuropsychiatric disorders and the duration in position for each employees, assessed with linear regressions. There is strong statistical association between insomnia and duration in position (R^2^=91,83%), excessive daytime sleepiness and duration in position (R^2^=81,23%), and anxiety and duration in position (R^2^=87,46%). The relation between depression and duration in position was moderate (R^2^=69,14%). Figure 3 showed the prediction of the same neuropsychiatric diseases above by the socioeconomic status (pSES). A moderate relation between insomnia and pSES (R^2^=62,04%) and excessive daytime sleepiness and pSES (R^2^=53,62%) was found in Figures 3A and 3B. The Figures 3C and 3D showed a strong association between anxiety and pSES (R^2^=82,95%), and between depression and pSES (R^2^=89,77%).

**Figure 4:**
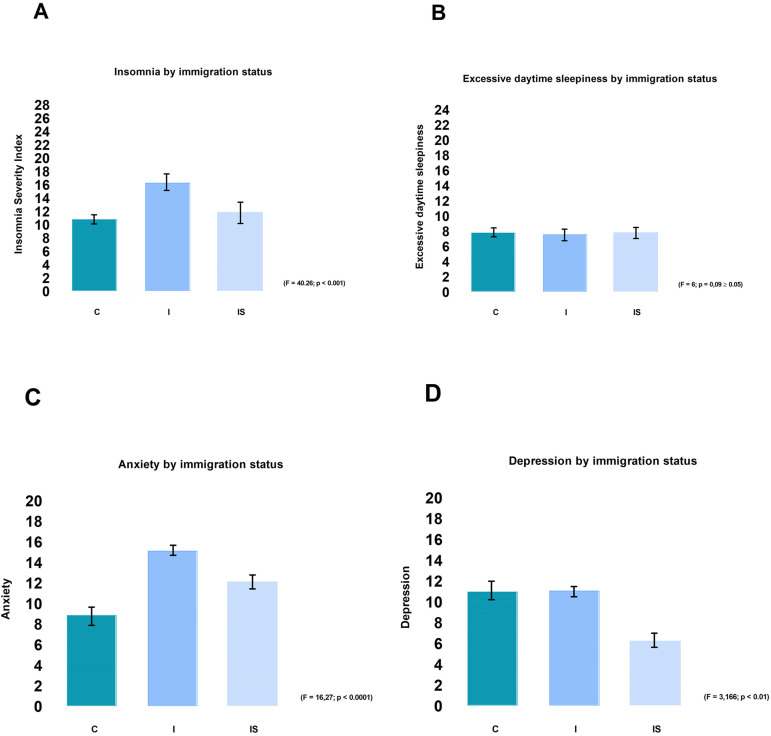
**Distribution of neuropsychiatric diseases among the subgroups of Canadian-born, immigrants and international students**. A one-way ANOVA with a Tukey multiple comparisons of means was performed to analyze differences between citizens (C), international students (IS) and immigrants (I) in their level of anxiety, depression, insomnia and excessive daytime sleepiness. Confidence interval:95%, α:0.05

Figure 4 reported the results of the ANOVA performed to assess differences between citizens, international students and immigrants in their level of anxiety, depression, insomnia and excessive daytime sleepiness. Figures 4A and 4C showed that being an immigrant is associated with clinical insomnia and more strongly with higher levels of anxiety, followed by the status of international student and lesser by Canadian-born (respectively F=40.26, *p<*0.001; Post Hoc, *p*<0.001 and F=16.27, *p*<0.001; Post Hoc, *p*<0.001). Figure 4D showed a higher score of depression for Canadians and immigrants compared to international students (F=3.166, *p*<0.001; Post Hoc, *p*<0.001). Analysis did not find a significant difference in excessive daytime sleepiness for the three sub-groups (F=6; *p*=0.09 >0.05; Post Hoc, *p*<0.001).

The trends of the neuropsychiatric diseases in the entire cohort and the differences between full-time and part-time workers, and regardless of gender, are shown in the Table 2.

**Table 2:** Comparisons of neuropsychiatric disorders between the full-time workers and the part-time workers in the entire cohort.

Neuropsychiatric diseases (tests/questionnaires)	Men			Women		
	Full time employed	Part-time employed	p-value	Full time employed	Part-time employed	*p*-value
Insomnia (Insomnia Severity Index)	17/28	12/28	***	16/28	14/28	*
Sleepiness (Epworth Sleepiness Scale)	9/24	5/24	**	9/24	6/24	**
Anxiety (Hospital Anxiety and Depression Scale-A)	12/20	9/20	***	11/20	9/20	***
Depression (Hospital Anxiety and Depression Scale-B)	10/20	10/20	ns	9/20	9/20	ns

A: Difference in mean score of the Insomnia Severity Index. B: Difference in mean score of the Epworth Sleepiness Scale. C: Difference in mean score of the Hospital anxiety and depression scale, part A (HADS-A). D: Difference in mean score of the Hospital anxiety and depression scale, part B (HADS-B). *p* < 0.001(***), *p* < 0.01 (**), *p* < 0.05 (*), ns (non-significant)

## DISCUSSION

The present research was led to document the epidemiology of neuropsychiatric diseases among employees working in customer service. Considering results above, the three objectives were completed. First, insomnia and excessive daytime sleepiness were found in the sample, with statistics for men and women on one side; and for part-time and full-time workers on the other side. No previous study reporting insomnia and sleepiness for people working in the customer service was found until now in the literature, despite the similarities between shift work in call centers and other jobs such as pilots and nurses^16^^,^^25^^,^^26^, it is one of the main strengths of this paper.

Data were reported on cardiovascular and metabolic diseases of people working in sector with rotating shift^14^ and some others showed side effects of irregular shift on allostatic load and mood disorders^14^^,^^27^^,^^28^. These studies reported that some work with rotating shifts like oil refinery operators or nurses, were associated with changes in the metabolism of hormones like melatonin, prolactin, testosterone and cortisol without any apparent phase shift of these hormones^27^. Further evidence also demonstrated that diurnal rhythms in cortisol, melatonin and HRV are not adapted to night work after 1-3 consecutive night shifts^29^. When a worker experienced during several months an irregular shift work, he finally developed a circadian disruption which was recently recognized as a risk factor for diabetes, cancer and poor cardiovascular outcomes^28^^-^30. Additional evidence has also linked shift work with sleep disorders, with a proportion estimated by the 2005 International Classification of Sleep Disorders around 2 to 5% of night shift workers^31^. It is in line with previous studies which stated that individual tolerance to irregular shift work was a complex issue influenced by the duration in position, the chronotype preference and the genetic background^29^^,^^31^^,^^32^. Few sleep disorders like excessive daytime sleepiness appear during intermittent night shifts and they are related to desynchronization of the circadian rhythms, disturbing attention and vigilance of the workers after an unknown period^29^^,^^31^. Obviously, the resulting shift work sleep-wake disorder affects the quality of life of the worker by creating cognitive decline or mood disorders^14^. Regarding anxiety and depression, few studies reported the impact of type of work on neuropsychiatric outcomes^33^^,^^34^.

The present study is the first which reports prevalence and proportion of anxiety and depression among customer service employees, and the first to describe a strong relation between perceived socioeconomic status and mood disorders in line with few articles reporting possible relation between socioeconomic status and health^33^^,^^35^^-^43. The present study alerts on the potential effect of working full-time in a call center as a risk factor for neuropsychiatric illnesses, regardless of age which was controlled in our regression models.

According to the findings, people working full-time obtained the highest score in the different questionnaires assessing anxiety, depression, insomnia and excessive daytime sleepiness. A higher score indicates a poor diagnosis for all the diseases evaluated, and even if the current transversal design did not allow to follow the impact of working full-time longitudinally on sleep disorders, anxiety and depression; findings presented here are trustworthy pictures of the mental health of the participants who are mainly customer service advisors. Taking into account presence of rotating shift and irregular shift work in customer service, these findings document an existing association between working full-time in customer service and neuropsychiatric diseases. This association was confirmed this year by a recent Japanese cross-sectional study performed with 18,682 participant full-time workers (7,098 female and 11,584 male) which confirmed an association between long working hours and sleep disorders, with an increase in cognitive impairment among bad sleepers^15^. The results also question the role of the duration in position and socioeconomic status as risk factors for neuropsychiatric diseases for customer service employees. Duration in position can be influenced by age. In order to avoid this bias, age was controlled in the linear regression analysis which is also another strength of this study. While social factors and sociodemographic factors may influence health in general^2^^,^^42^^-^46 and even if evidence exists on their key role as mediators between socioeconomic status and health^35^^,^^36^^,^^46^^-^50, nobody really investigated how people working in such fields are exposed to chronic stress, and current literature contains no data on employee’s health in this sector. The present paper draws an interesting profile of employees in terms of age, education, socioeconomic status, immigration status and duration in position in customer service in Canada. This is another strength of the present article, because no other study examined so deeply this population who is probably also exposed to shift work sleep-wake disorders and many other neurological disorders commonly found in similar employments^29^^,^^31^.

Despite previous questions raised by these results and debated in the previous paragraphs, some limitations exist for this paper. First, there is the difficulty in knowing what the exact role of each participant is in their respective companies, and this may be a limit because the level of stress experienced by a manager is different from the one experienced by an advisor, as showed by few authors who documented the difference in daily stress of cortisol secretion due to some socioeconomic markers like education, occupation or income^34^^,^^36^^,^^51^. In other words, someone with a high socioeconomic status (like a white collar) will have most often less stress and lower levels of cortisol compared to with an individual with a low socioeconomic status (like a blue collar)^50^^,^^52^^,^^53^. The present study did not investigate this difference. More research is necessary to produce more details about the influence of an individual’s position in a company on his/her health outcome.

Another limit found here was the absence of medical history or confounding factors like body mass index and respiratory disease. It was reported that excessive daytime sleepiness is often associated with sleep apnea, which can be mistaken for snoring, which is strongly associated with obesity^54^^,^^55^. Some treatments for anxiety may induce insomnia^54^ and some participants taking these kinds of medication may have been included in this study. Even if this has no influence on the study’s objectives and results, further studies should pay attention on this possible bias, which may affect research where mental disorders, or any other diseases studied, are linked to medical history or can be influenced by medication. In the same order, subscales of the questionnaire were not analyzed in this case only because it was not necessary for this pilot study; but it will be an improvement if further research investigates the current findings to see if the same trends arise when looking at sleep quality, sleep satisfaction or other components based on a different sleep questionnaire such as PSQI.

For now, here are some practical suggestions to reduce neuropsychiatric disorders for customer service employees or to at least mitigate the work burden on their brains:

***Avoid rotating shift as much as possible:*** As explained previously, the stabilization of an individual’s circadian rhythm is difficult with an irregular sleep and a fast rotating shift^14^^,^^56^. It is better to recruit employees working only night shifts or those working only day shifts. Changing shifts of an individual will create more cost for the employer because of the risk of employees developing poor health outcomes. In the present study, neuropsychiatric diseases were investigated but it may be possible that the same employees have musculoskeletal diseases, metabolic disorders or burn-out, which were already reported as side effects of rotating shift^14^^,^^56^.

***Encourage physical activity during a shift:*** Many studies reported the benefits of moderate physical activity on health, whatever type of activity it is^57^^-60^. It is true for elderly, children or adolescents. It is not a waste of money to extend the lunch time during a full shift, to allow people to walk somewhere, do a short yoga session or bike^57^^-60^.

***Increase part-time shift:*** As was demonstrated in this study, people working full-time reported much more neuropsychiatric diseases than part-time workers. According to the diversity of the Canadian population and the different subgroups of participants in this study, the mental disorders found here are not related to ethnicity, age or some bias; the sample is very representative of the general population. This also means that results are clearly a warning on the dangers related to the stressful environment that is customer service. In my opinion, reducing full shifts to 6 or 7 hours including a 30 minute-lunch break may be a good compromise for both the employers and the employees in terms of cost, to decrease the number of sick day-related absences and to increase work performance.

***Enlarge the investigation to other countries:*** The Canadian population is similar to those of the United Kingdom, the United States of America, France, Belgium, Australia and Germany because of similar immigration patterns. Customer service is an important part of their economy as industrialized countries, so, without knowing it, these countries probably face the same neuropsychiatric disorder problems with their employees. More research in these countries may be interesting and informative.

In conclusion, customer service employees are exposed to a continuous stimulation of their cognitive functions in addition to different stressors which can progressively and silently affect mental health and performance at work. Investigations on mental health and physical health of customer service employees are worthy of interest for a wide audience interested in occupational health and safety. The topic should also be explored by people planning to work in this field; and companies which want to decrease long sick leaves and increase staff performance. Future investigations (which hopefully will be longitudinal) should integrate participants’ medical records and different types of shifts available in their framework. This would be done to analyse if the neuropsychiatric diseases reported here are influenced by night shifts or by a specific schedule. The next research should also document the impact of the individual’s chronotype regarding the association between sleep disorders and a long time working in call centers. The chronotype should also be further analysed in the relation between mood disorders and working full-time versus part-time.

## CONCLUSION

The present study alerts on the potential effect of working full-time in a call center as a risk factor for neuropsychiatric illnesses. Customer service employees are exposed to a continuous stimulation of their cognitive functions in addition to different stressors which can progressively and silently affects the nervous system. It also reveals that employees working full-time with rotating shifts are more exposed to insomnia, sleepiness and anxiety than employees working part-time. More research is needed to understand mental health of customer service employees regardless of their area and it is worthy of interest to study the link between sleep disorders and mood disorders with work conditions.
